# Slow Freezing, but Not Vitrification Supports Complete Spermatogenesis in Cryopreserved, Neonatal Sheep Testicular Xenografts

**DOI:** 10.1371/journal.pone.0123957

**Published:** 2015-04-29

**Authors:** Budhan S. Pukazhenthi, Jennifer Nagashima, Alexander J. Travis, Guilherme M. Costa, Enrique N. Escobar, Luiz R. França, David E. Wildt

**Affiliations:** 1 Center for Species Survival, Smithsonian Conservation Biology Institute, National Zoological Park, Front Royal, Virginia, United States of America; 2 The Baker Institute for Animal Health, College of Veterinary Medicine, Cornell University, Ithaca, New York, United States of America; 3 Atkinson Center for a Sustainable Future, Cornell University, Ithaca, New York, United States of America; 4 Laboratory of Cellular Biology, Department of Morphology, Federal University of Minas Gerais, Belo Horizonte, Minas Gerais, Brazil; 5 Department of Agriculture, Food and Resource Sciences School of Agriculture and Natural Sciences, University of Maryland-Eastern Shore, Princess Anne, Maryland, United States of America; University Hospital of Münster, GERMANY

## Abstract

The ability to spur growth of early stage gametic cells recovered from neonates could lead to significant advances in rescuing the genomes of rare genotypes or endangered species that die unexpectedly. The purpose of this study was to determine, for the first time, the ability of two substantially different cryopreservation approaches, slow freezing versus vitrification, to preserve testicular tissue of the neonatal sheep and subsequently allow initiation of spermatogenesis post-xenografting. Testis tissue from four lambs (3-5 wk old) was processed and then untreated or subjected to slow freezing or vitrification. Tissue pieces (fresh, n = 214; slow freezing, then thawing, n = 196; vitrification, then warming, n = 139) were placed subcutaneously under the dorsal skin of SCID mice and then grafts recovered and evaluated 17 wk later. Grafts from fresh and slow frozen tissue contained the most advanced stages of spermatogenesis, including normal tubule architecture with elongating spermatids in ~1% (fresh) and ~10% (slow frozen) of tubules. Fewer than 2% of seminiferous tubules advanced to the primary spermatocyte stage in xenografts derived from vitrified tissue. Results demonstrate that slow freezing of neonatal lamb testes was far superior to vitrification in preserving cellular integrity and function after xenografting, including allowing ~10% of tubules to retain the capacity to resume spermatogenesis and yield mature spermatozoa. Although a first for any ruminant species, findings also illustrate the importance of preemptive studies that examine cryo-sensitivity of testicular tissue before attempting this type of male fertility preservation on a large scale.

## Introduction

While most research has been directed at common ungulates (cow, pig, sheep, goat), hoofed animals represent one of the most diverse, higher order taxonomic groups with a worldwide distribution [1]. There are more than 450 ungulate species comprising two orders, the Artiodactyla (animals with a cloven hoof) and Perissodactyla (those with an odd number of toes). Our laboratories are keen to bring attention to these understudied species, characterize their fundamental reproductive biology and, most importantly, develop approaches for preserving their genetic integrity and heterozygosity. Virtually all of these species are non-domesticated and vulnerable to further population declines or even extinction due to habitat loss and persecution, especially hunting [2]. More than 180 wild ungulate species (~40% of the total) are maintained in zoological collections for (1) exhibition, (2) managed reproduction to retain existing gene diversity and (3) as insurance populations for their wild counterparts living in nature [3]. Recent evidence, however, suggest that more than half of all ungulate populations managed in captivity are not self-sustaining, that is, more animals are dying than being born [4]. The issue is exacerbated by too few genetically valuable individuals in zoos (often due to low founder numbers), a 15 to 20% neonatal loss within the first year of birth and overall declining available space, in part, because of a perceived lack of public interest in ungulates compared to more charismatic species [4–6].

Because at least one in six ungulate calves born in zoos dies before reaching reproductive age [7,8], there appears to be a conservation opportunity—preserve testicular tissue from young males in a way that would allow reinitiation of spermatogenesis and the eventual recovery of viable spermatozoa. A basis for this justification comes from work in common, mostly domesticated species where testicular tissue is recovered within 24 h of death and the testicular parenchyma dissected into 1 to 2 mm^3^ pieces that are placed immediately (or after cryopreservation/thawing) under the skin of special, immune-suppressed mice to initiate spermatogenesis *in vivo* [9–10]. Round spermatids, elongating spermatids or spermatozoa have been isolated from these xenografts and used with intracytoplasmic sperm injection (ICSI) to produce embryos or live offspring [9–11]. With fresh testicular tissue, this approach has been used to achieve complete or advanced stages of spermatogenesis in the domesticated goat [12], pig [12], rabbit [13], bull [14,15], cat [16,17], horse [18], sheep [19,20], dog [21], hamster [22], ferret, *Mustela putorius* [23] and the non-domesticated bison (*Bison bison bison* [24], rhesus monkey [25], Javan banteng (*Bos javanicus javanicus* [26], white-tailed deer (*Odocoileus virginianus* [27]), Mohor gazelle (*Gazella dama mohor* [28]) and collared peccary (*Tayassu tajacu* [11]) as well as the human [29]. Advanced stage embryos and/or live offspring (mice [22]; pig [30–33]) have been produced via ICSI with sperm derived from xenografts.

To date, complete or advanced stage spermatogenesis after testicular tissue thawing and xenografting has been reported for only two species, the mouse [22,34–36] and pig [37–39]. Live offspring also have been produced following ICSI with spermatozoa derived from thawed xenografts from these same two species (mouse [40]; pig [32,41]). Success has been more elusive after xenografting thawed testicular tissue in the cat [42], human [43,44], Iberian lynx (*Lynx pardinus* [28]), Cuvier’s gazelle (*Gazelle cuvieri* [28]), Mohor gazelle [28], Indian white spotted mouse deer (*Moschiola indica* [45]) and rhesus monkey [46,47]. For example, in previously cryopreserved, human testicular tissue, spermatogenesis failed to progress beyond the pachytene stage after orthotopic grafting [29,47,48], whereas there was a complete loss of germ cells in the cat [42]. Similar studies in the Cuvier’s gazelle and Iberian lynx also were discouraging with only gonocytes and spermatogonia surviving, respectively, after xenografting [28].

Although xenografting of fresh tissue often yields resumption of spermatogenesis and production of elongating spermatids/spermatozoa, this approach generally is impractical in the real world due to lack of available immuno-suppressed recipient mice for the transplantations. Although cryopreservation can be a solution, the conditions that preserve structural integrity of the tissue often lead to anomalies in cellular function after xenografting [29,44,48]. To date, testicular tissue from various mammals has been cryopreserved using slow freezing or vitrification [13,22,28,29,34–52], one approach or the other. For the former, tissue pieces are equilibrated in a comparatively low concentration (0.2–0.3 M) cryoprotectant and then frozen gradually at ~1°C/min in a -80°C freezer overnight before plunging and storing in liquid nitrogen. Alternatively, vitrification involves converting the biomaterial into a glass-like, amorphous solid without triggering ice crystal formation [53,54]. Vitrification is characterized by short-time exposure to high concentrations (0.5–5 M) of usually multiple cryoprotectants and then plunging/storing directly in liquid nitrogen [53].

It has been unclear why the fertility preservation approach involving freezing-thawing and then xenografting of testicular tissue has been effective for two species (mouse, pig), but not for others tested. Our hypothesis was that neonatal tissue was sensitive to cryo-approach and that conducting preemptive, comparative studies focused on preservation conditions could mitigate structural and functional damage and facilitate achieving spermatogenesis post-grafting. We chose the neonatal sheep as a model because there were limited studies in ruminants, and we expected that lessons learned would be applicable for future studies directed at prepubertal ungulates of rare genotypes and endangered species. Our goal was to determine the influence of cryopreservation conditions, specifically slow freezing versus vitrification, on preserving testicular tissue from the neonatal lamb. Success was determined by evaluating the influence of treatment on ability to support spermatogenesis after tissue transplantation into immuno-suppressed mice.

## Materials and Methods

### Chemicals

Unless otherwise indicated, all chemicals were purchased from Sigma-Aldrich Chemical Company (St. Louis, MO).

### Animals

Animal procedures were reviewed and approved by the Animal Care and Use Committees of the Smithsonian Conservation Biology Institute and Cornell University. Four 3 to 5 wk old male, Katahdin or Kathadin x Dorper cross lambs from the University of Maryland—Eastern Shore were the source of testes post-castration. Freshly-excised testes were immersed immediately in TCM 199 and transported in a 5°C cold box to the Smithsonian Conservation Biology Institute (Front Royal, VA) for processing. Testes averaged 2.01 ± (standard deviation) 0.1 cm in length by 1.41 ± 0.2 cm in width, with a mean weight per testis of 2.08 ± 0.2 g. For control, fresh tissue xenografting, one random testis from each donor was shipped overnight on ice to the Baker Institute, Cornell University (Ithaca, NY) where transplantations (see below) were conducted within 24 h of original excision. The contralateral testes were processed (as described below) before storage in liquid nitrogen. On average, we generated 183 grafts (range, 156–210) from each pair of testes. Xenograft recipients were 4 wk old SCID mice (n = 49; Taconic, Germantown, NY) that were housed (12:12 h light:dark cycle) at Cornell University inside a class 100 cabinet in sterilized caging on Aspen Chip Bedding (P. J. Murphy Forest Products, Montville, NJ). The cabinet was maintained at 22 to 24°C, with 40 to 70% relative humidity and 10 to 12 air exchanges hourly. Irradiated diet (ProLab IsoPro 3000; LabDiet, St. Louis, MO) and autoclaved, acidified water were provided *ad libitum*.

### Tissue processing and cryopreservation

Testes were washed three times in fresh HEPES-buffered DMEM supplemented with pyruvate (1 mM), glutamine (2 mM), penicillin (10,000 IU/mL), streptomycin (10 mg/mL) and 20% (vol/vol) heat-inactivated, fetal bovine serum (Handling Medium, HM). The tunica albuginea was excised and the testis dissected into small (~1 mm^3^) pieces. Four to five pieces of fresh tissue per donor were fixed overnight in Bouin’s fluid and then transferred to 70% ethanol until processing for histology and assessment of developmental stage. For cryopreservation, remaining tissue pieces from each testis were divided equally with half preserved using the slow freezing method [38] and the other half by vitrification [55].

For the slow approach, freezing medium consisted of heat-inactivated fetal bovine serum, DMEM with HEPES and dimethylsulfoxide (DMSO) at a ratio of 1:3:1 [38]. Each 2.0 mL cryovial (Fisher Scientific, Pittsburgh, PA) contained eight to 10 testicular tissue pieces immersed in 0.5 mL of the freezing medium at room temperature (21°C). Each cryovial then was placed into a Nalgene (Mr. Frosty, Fisher Scientific) freezing container with isopropyl alcohol at room temperature that then was transferred into a -80°C freezer overnight ([35], Revco, Fisher Biotech). This unit is designed to provide a cooling rate of ~-1°C min^-1^. The following morning, all cryovials were removed from the unit and plunged into a liquid nitrogen-containing dewar and stored for 8 to 16 wk before xenografting. The slow frozen vials were removed from liquid nitrogen, held at room temperature for 1 min (to evaporate liquid nitrogen remaining in the container) and then immersed in a 25°C water bath for 1 min. Two mL of HM were added immediately to each vial and the contents transferred to a sterile Petri dish. After 2 min, tissue pieces were transferred to a fresh dish containing 5 mL of HM for a 5 min period of gentle swirling incubation, a process that was repeated two additional times [38]. These thrice washed pieces were assessed immediately for cell viability (see below), histological assessment (fixation in Bouin’s fluid) or processed for xenografting (see below).

For vitrification, tissue pieces first were incubated at room temperature in HM containing 7.5% (vol/vol) each of ethylene glycol (EG) and DMSO for 25 min followed by a second equilibration in HM containing 20% (vol/vol) each of EG and DMSO for 15 min [55]. Excess fluid was removed by dabbing each piece on sterile gauze and then placing on 4 cm long strips of aluminum foil that were submerged directly using tongs into a 30 cm deep liquid nitrogen batch. Within 10 min, the aluminum strips were transferred into 4.5 mL labeled cryovials (3 strips/vial) and stored in liquid nitrogen for 8 to 16 wk. For thawing, aluminum strips containing tissue pieces were removed from each cryovial while immersed in liquid nitrogen and then transferred immediately into a Petri dish containing 10 mL of Solution 1 (HM containing 1 M sucrose) at 37°C with constant swirling. This step released the pieces from each aluminum foil strip. After a 1 min equilibration, the pieces were transferred rapidly into a second dish containing Solution 2 (HM containing 0.5 M sucrose), this time at ambient temperature for 5 min. All pieces then were washed twice in 5 mL MH each time by gentle swirling (10 min, ambient temperature). These warmed pieces were assessed immediately for cell viability (see below) or histology or processed for xenografting (see below).

### Cell viability assessments

Cell viability was determined by enzymatic digestion of tissue pieces to a single cell suspension followed by staining with SYBR-14 and propidium iodide (Cell Viability Kit, Invitrogen, Grand Island, NY [56]). Briefly, tissue fragments were digested successively at 37°C with collagenase (2 mg mL^-1^ in DMEM) for 30 min, hyaluronidase (3 mg mL^-1^ in DMEM) for 15 min, trypsin-EDTA (0.0005 g trypsin plus 0.00025 g EDTA mL^-1^ in PBS) for 1 min and deoxyribonuclease (7 mg mL^-1^ in DMEM) for 1 min. The final cell pellet was washed by centrifugation (300 x *g*; 5 min) in fresh DMEM containing 5% FBS, and a 10 μl aliquot of cell suspension was stained with SYBR-14 (0.01 mg mL^-1^) and PI (9.6 μM) for 15 min at 37°C in the dark. Cells were washed by centrifugation (300 x *g*; 5 min) in 0.5 ml of fresh DMEM, and at least 200 cells per sample were counted as either live (SYBR-14 positive cells, green fluorescence) or dead (PI positive cells, red fluorescence) using epifluorescence (BX 41; Olympus Corporations, Center Valley, PA).

### Xenografting of testicular tissue pieces into mice and graft recovery

Xenografting of fresh and cryopreserved tissue was performed at the Cornell University laboratory as described previously [17]. All tissue fragments from different conditions, that is, fresh (18 mice, 214 grafts), uncontrolled slow (15 mice, 210 grafts) or vitrification (16 mice, 222 grafts) were xenografted to different recipient mice. Briefly, anesthesia was induced and then maintained in the male SCID mice using 1.5 to 3.5% isoflurane gas. Castration was performed via a midline abdominal approach to eliminate endogenous gonadal hormone influence. Then, three to six, 1 cm long incisions were made in the skin over the back to insert 11 to 14 tissue pieces (xenografts) bilaterally (5–7 pieces per side). A 6–0 silk (Ethicon, Somerville, NY) suture was placed to mark the site of each graft to facilitate subsequent piece recovery. The incisions were closed with skin staples (Braintree Scientific, Braintree, MA) and an i.m. injection of buprenorphine (Bedford Laboratories, Bedford, OH; 1 mg kg^-1^) provided for analgesia. For graft recovery, each mouse was euthanized by cervical dislocation 17 wk after graft placement. A dorsal skin incision was made to expose the grafts that were excised and immediately immersed in Bouin’s fixative solution.

### Assessment of xenograft volume density, seminiferous cord and tubule number and germ cell development

Testicular parenchymal volume density was determined using images captured by light microscopy using a 540-intersection grid from ImageJ Software (National Institutes of Health, http://rsb.info.nih.gov/ij/). Points on the grid associated with the images were classified as part of the tubular compartment (comprising tunica propria, epithelium and lumen) or intertubular compartment (Leydig cells, connective tissue and blood vessels). Fifteen randomly chosen fields per image (8,100 points) were scored for each xenograft (fresh, n = 96; slow freezing and then thawing, n = 80; vitrification and then warming, n = 42) at 200x. Number of points per testicular compartment was divided per 8,100 points in each xenograft to achieve the testicular volume density and expressed as a proportion. To determine progression of spermatogenesis, all seminiferous cord and tubule cross-sections present per image were examined for the most advanced germ cell type present [57]. The seminiferous cords were those distinctive structures within the tissue that had no central lumen, but contained immature Sertoli cells and some germ cells. By contrast, the tubules had a well-defined lumen associated with mature Sertoli cells and more germ cells.

### Statistical analysis

Data were tested for normality using a Shapiro-Wilk normality test. Parametric data were evaluated by analysis of variance and differences compared by Tukey’s testing. Non-parametric analyzes were conducted using Kruskall-Wallis followed by Dunn’s multiple comparison tests. All analyses were performed using the GraphPad Prism 5 (GraphPad Software, Inc., La Jolla, CA) or SAS (Cary, NC). All data were expressed as mean ± standard deviation values and differences considered significant at *P*<0.05.

## Results

### Testicular morphology and total cell viability before and after cryopreservation

**Fig 1:**
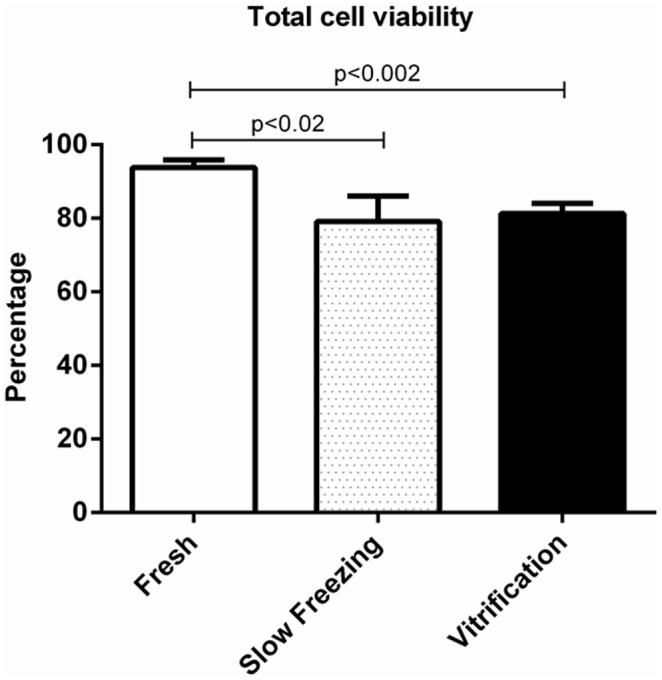
Total cell viability of neonatal lamb testis tissue immediately after castration (fresh), cryopreserved by slow freezing and vitrification. Values represent mean ± SD of three donors (three replicates per donor). Lines above the error bars denote significance.

Total cell viability after short time storage (18–22 h) at 4°C (control) exceeded 90% (Fig 1). Following either slow freezing or vitrification, total cell viability was reduced, but comparable to each other at ~80% (Fig 1).

**Fig 2:**
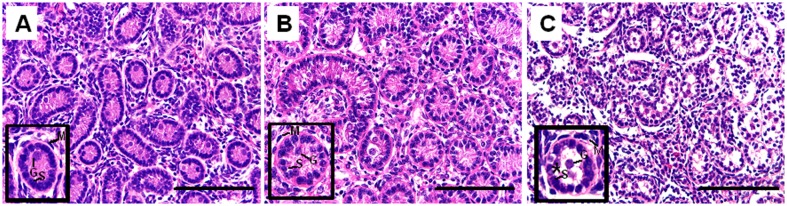
Histological appearance of immature lamb testis tissue exposed to one of three conditions. (A) control (fresh), (B) slow freezing and then thawing or (C) vitrification and then thawing (400x). Inset in each panel depicts the seminiferous cords (SC) at a higher magnification. Slow freezing preserved normal SC integrity similar to the fresh control, whereas vitrification caused disruptions, including evidence of shrinkage around the SC (*). G = gonocyte, S = Sertoli cell, M = peritubular myoid cell. Scale bar = 100 μm.

After storage and at the time of grafting, the tissue pieces were comprised of seminiferous cords containing virtually only Sertoli cells, gonocytes and interstitial tissue (Fig 2A). Structural integrity and architecture were comparable between the fresh control and the slow freezing and thawing groups as revealed in Fig 2A and 2B. By contrast, thawed vitrified samples displayed moderate disruptions, including shrinkage of Sertoli cells within the seminiferous cords and an increase in interstitial space indicative of freeze-thaw induced damage (Fig 2C).

### Testis graft survival and growth after xenografting

Testis graft survival was based on number of viable grafts recovered per number originally transplanted. Failed grafts were those that atrophied between the time of placement and recovery. At 17 wk after transplantation, testis graft survival was comparable for fresh (45.8 ± 11.4%) and slow freezing (41.3 ± 18.1%) groups, but less (*P*<0.05) post-vitrification (30.1 ± 9.2%).

### Histological evaluation of testicular tissue xenografts

**Fig 3:**
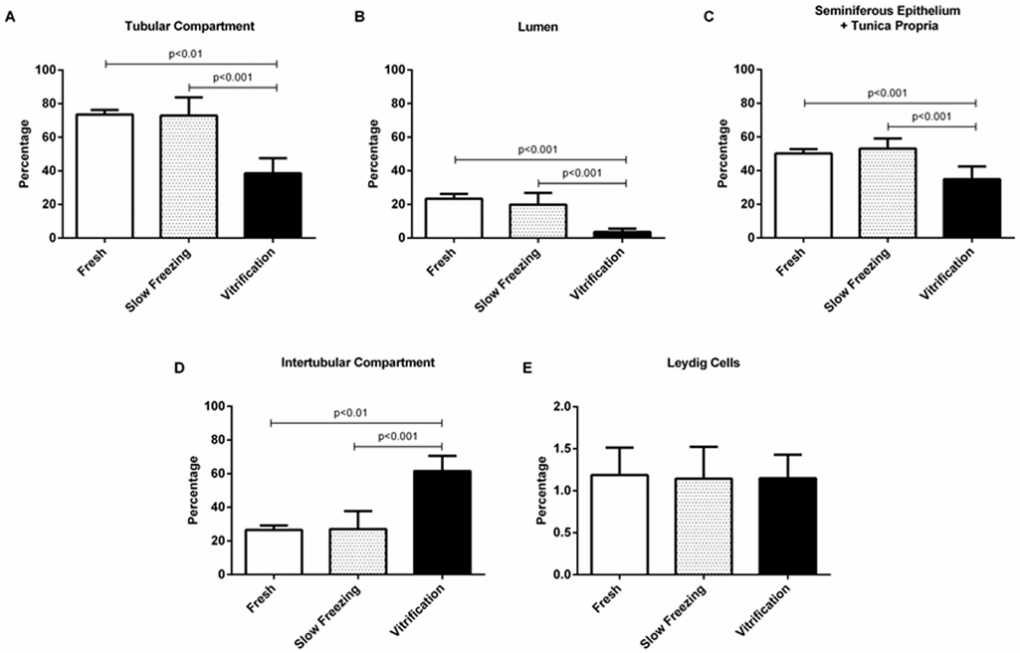
Xenograft volume density traits for pieces of lamb testis transplanted fresh (control) versus after slow freezing and thawing versus vitrification and thawing. For each assessed metric, data for the slow freezing group were no different (*P*>0.05) from the control. Within a trait, lines above the error bar denote significance.

**Fig 4:**
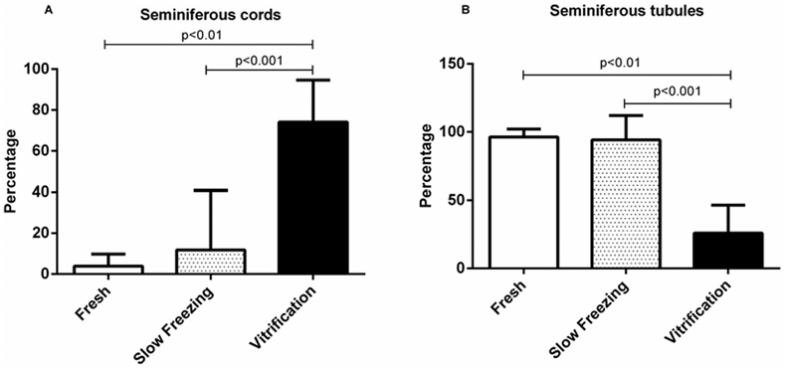
Relative percentage of seminiferous cords versus tubules in cross-sections of lamb testis pieces that were retained fresh versus those that were subjected to slow freezing and warming versus those that were vitrified and thawed, with all then xenografted into SCID mice. Testis fragments exposed to slow freezing were no different from controls (*P*>0.05) in contrast to a preponderance of only unchanged seminiferous cords in the vitrified group (Panel A). Within a trait, lines above the error bar denote significance.

Histological assessments of tissue grafts at 17 wk revealed more area comprised of tubular compartment, tubular lumen, seminiferous epithelium and tunica propria in fresh and slow frozen samples compared to vitrified counterparts (Fig 3A–3C). By contrast, intertubular compartment values were higher in vitrified compared to fresh (*P*<0.01) or slow frozen tissue (*P*<0.001; Fig 3D). The proportion of tissue volume comprised of Leydig cells did not differ among the three treatments (Fig 3E). Fresh and slow frozen tissue xenografts also expressed similar percentages of seminiferous cords (Fig 4A) and seminiferous tubules (Fig 4B). Xenografts derived from vitrified tissue retained mostly unchanged seminiferous cords with few seminiferous tubules.

**Fig 5:**
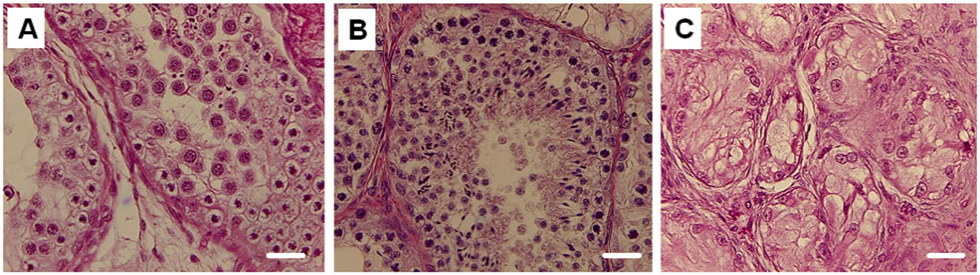
Morphology of lamb testis pieces that were retained fresh (A) versus those subjected to slow freezing and thawing (B) versus those that were vitrified and thawed (C). All then were xenografted into SCID mice before excision and evaluation at 17 wk. Sections were stained with hematoxylin-eosin. Each scale bar = 100 μm.

Representative histological images from each of the treatment groups of recovered xenografts at 17 wk are depicted in Fig 5. Complete spermatogenesis, including round and elongated spermatids, were detected in fresh (Fig 5A) and slow frozen (Fig 5B) groups, but not after vitrification (Fig 5C). By contrast, spermatogenesis in the latter group appeared impaired on the basis of a decrease in tubular compartment and lumen space and a corresponding increase in inter-tubular space. For this treatment, the most advanced spermatogenic cells were only a few primary spermatocytes.

**Fig 6:**
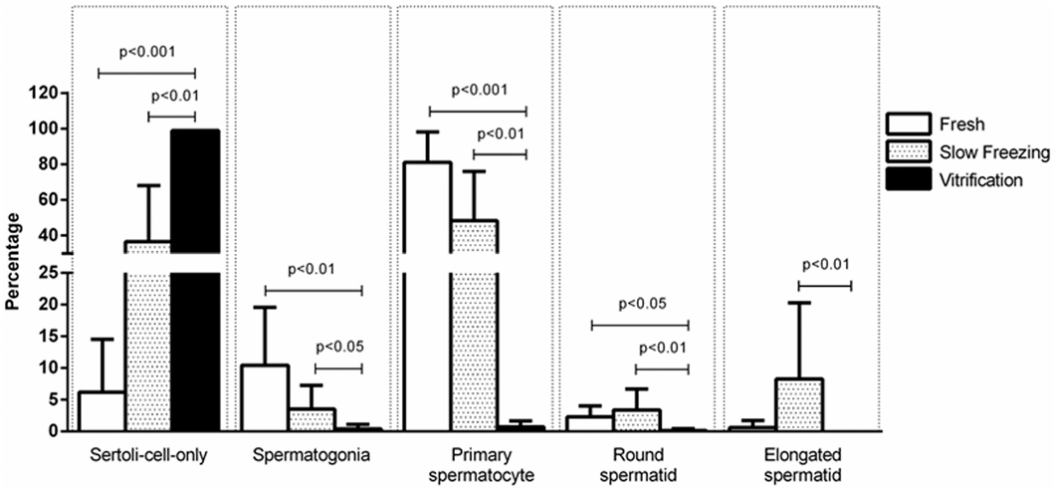
Prevalence of advanced germ cell type per seminiferous cord/tubule cross-sections in xenografts that originally were fresh controls versus those subjected to slow freezing versus vitrification. The latter treatment resulted in production of mostly spermatogonial cells and primary spermatocytes in contrast to slow freezing where few immature germ cells remained along with a higher proportion of more advanced cell types, including elongated spermatids. Within a trait, lines above the error bar denote significance.

In a more detailed assessment of cell types, most significant was that >90% of seminiferous tubules after vitrification were comprised of Sertoli cells only compared to ~40% for slow freezing or ~6% for fresh controls (Fig 6). Proportions of spermatogonia and primary spermatocytes were higher in fresh tissue and after slow freezing with proportions negligible post-vitrification (Fig 6). Approximately 5% and 10% of seminiferous tubules in slow frozen xenografts contained round and elongated spermatids, respectively (Fig 6). Similarly, approximately 3% and 1% of seminiferous tubules in the fresh controls contained round and elongated spermatids, respectively. There were no differences in these values between fresh and slow frozen tissues, with proportions of both being higher than for vitrified counterparts (*P*<0.05 and *P*<0.01, respectively).

## Discussion

There is a need to develop fertility preservation protocols for diverse species, all of which are producing surplus germplasm, most of which never contributes to actual reproduction [28,58–62]. There are few practical options for rescuing the germline of immature, prepubertal individuals. One exciting approach for males is grafting a donor’s fresh testicular tissue under the skin of an immuno-suppressed mouse to generate mature spermatozoa capable of fertilization [9,10,12]. For this approach to have widespread, practical application, there is a need to be able to store the fresh tissue for indeterminate intervals before transplanting to a homologous or heterospecific recipient. With storage capability, there is potential for salvaging the reproductive genome of any male of any age as long as the material is collected in relative fresh condition. This ability would eliminate the need for maintaining on-site, expensive, specialized, recipient mouse colonies and permit valuable tissue to be preserved essentially anywhere, including at remote field sites. However, to-date there are only a few reports demonstrating the potential of freeze-thawing neonatal testicular tissue and then provoking successful, advanced spermatogenesis after xenografting [34–39]. Although this strategy has worked for two species (mouse, pig), it has been unsuccessful for a host of others [28,40–45] and has contributed to significant structural and functional abnormalities in preserved testis tissue. Here we chose the neonatal sheep as a study subject because complete spermatogenesis had not yet been achieved using this fertility preservation approach in any ruminant species. Our experiences in previous diverse cryo-studies, ranging from gametes [60,63,64] to embryos [63,65,66], also had clearly demonstrated that freeze-thaw method was a primary factor in dictating the survival of sensitive cells to such extreme storage conditions. The present findings clearly extended the significance of cryo-approach selected to gonadal tissue, specifically showing that slow freezing of lamb testicular tissue was far superior to rapid vitrification in preserving cellular integrity and function after placement into the immuno-suppressed mouse. The result was the first ever production of elongated spermatids from cold-stored, neonatal lamb, testicular tissue that had been xenografted into another species. But also significant was affirming the value of preemptive studies that examine cell sensitivity to cryo-approach. We suspect that such an early, comparative tactic could improve the viability of frozen-thawed testicular tissue, including for those earlier studied species where spermatogenesis recovery has failed or been limited [28,40–45].

In our neonatal lamb study, the slow freezing method retained most of the structural and functional features of the testicular tissue post-xenografting. Approximately 40 to 50% of the xenografts survived, and the most advanced stage of spermatogenesis achieved was elongating spermatids. The timeline for germ cell advancement, including complete spermatogenesis within 17 wk of transplantation, aligned with the duration reported for fresh neonatal sheep testicular tissue [20]. In contrast to earlier studies [15,16,22], we found that xenografting fresh tissues resulted in fewer tubules with spermatocytes compared to frozen-thawed counterparts. This observation was analogous to findings of Abrishami et al. [39] who reported more seminiferous tubules with more advanced spermatogenesis in cooled compared to fresh pig testicular tissue. The latter authors speculated that the developmental potential of the tissue may be positively influenced by metabolic changes during storage that permit faster recovery from castration-associated hypoxic stress.

What was clear from our study was that cryo-approach had a major impact on xenografting success. Specifically, slow preservation of neonatal lamb testicular tissue was preferable. By contrast, use of high concentration cryoprotectants and quick freezing via vitrification (an effective method for oocytes and embryos [63,65,66]) failed to support tissue survival and cell development. Of course, testicular tissue is a complex mix of germ cells, Sertoli cells, Leydig cells, peritubular myoid cells, vasculature and interstitial connective tissue with the inclusive assemblage being far more challenging to successfully freeze than a single cell, such as a spermatozoon [67]. A major driver of survival to freezing is the permeability of target cells to cyroprotectant type and concentration as well cooling rate. Under subpar conditions for a cell’s innate physical and chemical properties (including permeability), the freezing protocol becomes lethal, usually from cryoprotectant-induced toxicity and/or structural damage caused by ice formation and lysis [68]. These risks are enhanced in the presence of a diverse assortment of different, yet integrated cell types. We suspect that the success derived from our slow freezing protocol (relying solely on DMSO) was related to an imposed, gradual exchange of water and cryoprotectant at a low temperature over a protracted interval. The limitation with our vitification approach probably was related to exposing the tissue to the cryoprotectants at ambient temperature, which likely accelerated structural and functional tissue and cell damage.

Both slow freezing and vitrification decreased cell viability, but with only minimal-to-moderate disruption of tissue architecture. Even so, and regardless of approach, overall viability of testicular cells was >80% after cryopreservation and thawing. This observation was in agreement with Fredrickx et al. [69] who reported that functional capacity of spermatogonial stem cells may be severely impaired during cryopreservation despite high cell viability after thawing. This incongruity appears related to the propensity for dead cells to breakdown rapidly during tissue digestion for cell staining, thereby leading to underestimates of proportion of non-viable cells [38]. The marked difference between slow freezing and vitrification also likely was related to tissue sample size, each piece being at least 1 mm^3^. This larger size probably makes these tissue pieces highly vulnerable to intracellular ice formation associated with vitrification, cooling and warming. While failing to meet any of our success criteria, vitrification warrants further evaluation, in part, because it is a quick process with applicability to field conditions. As vitrification efficiency is well known to be associated with cryoprotectant type and concentration [47], research priorities could include assessing the benefits of ethylene glycol, polyvinyl pyrrolidone and trehalose for supporting complete spermatogenesis in xenografts.

We were surprised that the proportion of grafts surviving (i.e., those persisting subdermally for 17 wk) was circa 50%, especially as others have reported rates of 60% or greater for the mouse [12,34,36,70], pig [12,39], goat [20] and sheep [20]. Interestingly, successful graft recovery also has been lower (37–50%) or highly variable (0–75%) in the prepubertal Cuvier and Mohrr gazelle and neonatal-to-adult Iberian lynx, respectively [28]. These variations among species and laboratories no doubt are related to developmental stage of the testis donor as well as damage incurred during tissue manipulation and processing. Although we were encouraged that more than half of the transplants could be recovered after >4 mo placement, our goal is to increase percentage of recoverable grafts, thereby increasing sperm production while most efficiently using the specialized SCID mice. High priorities for next steps are examining the influence of tissue piece size and uniformity as well as age of the prepubertal donor [9,17,39] on graft survival.

Our findings also confirmed the benefits of DMSO as a preferred cryoprotectant during slow freezing, preserving both tissue structure and developmental capacity in the lamb model. This same cryo-agent has been used with modest-to-good success in the mouse [13,34,36,70,71] and pig [39]. Keros et al. [51] reported that DMSO had value for freeze-thawing testicular tissue from prepubertal boys, all while retaining normal histological architecture, *in vitro* testosterone production and spermatogonia within seminiferous tubules. Yildiz et al. [36] recently have argued that DMSO’s advantage is its ability to preserve functional Leydig cells at levels that hormonally facilitate spermatogenesis.

Testis xenografting has been promoted as a fertility preservation tool for a variety of species, including prepubertal human patients diagnosed with cancer [9,32,72,73]. However, there is significant risk as malignant cells would be reintroduced back to the patient during transplantation. An alternative is cryopreserving testicular biopsies that later could be thawed and grafted into immuno-suppressed mice to achieve advanced stages of spermatogenesis. Another option is isolating spermatogonial stem cells that could be differentiated *in vitro* to produce round or elongating spermatids or spermatozoa. It also may be possible to achieve competent spermatogenesis totally *in vitro* in a controlled microenviroment. Recently, Sato et al. [74] successfully cultured testicular explants from the neonatal mouse and produced mature spermatozoa that were injected into mature oocytes to produce advanced stage embryos and then live pups. In theory, this more precise, low-scale method should reduce the risk of contaminating the combined germplasm with cancer cells. To our knowledge, there are no other reports on using a strictly *in vitro* approach for more complex, non-murine species. Of course, the advantage of this strategy would be eliminating dependence on laboratory animals as recipients for the testis explants. Regardless, having the ability to preserve gonadal tissue soon after death or excision in a way that sustains tissue viability and allows re-initiation of full spermatogenesis remains the priority.

In conclusion, our findings for the neonatal sheep demonstrate that slow cryopreservation in the presence of DMSO preserved testes tissue cell viability and supported complete spermatogenesis when xenografted into the recipient SCID mouse. As the first such successful report in a ruminant, we offer three recommendations, the first being that slow freezing approach could be used now to store testicular tissue from invaluable sheep genotypes with confidence that viable spermatozoa eventually could be recovered. Second, it would seem prudent to begin testing this same protocol in other ungulates, especially ruminants, hopefully to demonstrate cross-species adaptability and application. Such a finding would encourage and justify testicular tissue preservation in rare, prepubertal individuals that die unexpectedly with no other hope of contributing to reproduction. Finally, this study reaffirms the significance of type of cryo-technology for successfully preserving the male genome. For this reason, future efforts directed at preserving testicular tissue would be best served by first comparatively exploring the sensitivity of this gonadal tissue to cryo-approach.
